# Modeling the impact of social determinants on breast cancer screening: a data-driven approach

**DOI:** 10.3389/fmed.2025.1644287

**Published:** 2025-08-20

**Authors:** Guofang Ma, Miranda G. Scully, Jiahui Luo, Jiazuo H. Feng, Christine M. Gunn, Roberta M. diFlorio-Alexander, Anna N. A. Tosteson, Sally A. Kraft, Wesley J. Marrero

**Affiliations:** ^1^Department of Biomedical Data Science, Geisel School of Medicine at Dartmouth, Lebanon, NH, United States; ^2^Department of Computer Science, Dartmouth College, Hanover, NH, United States; ^3^Thayer School of Engineering, Dartmouth College, Hanover, NH, United States; ^4^Dartmouth Cancer Center, Dartmouth Hitchcock Medical Center, Lebanon, NH, United States; ^5^Department of Medicine, Geisel School of Medicine at Dartmouth, Lebanon, NH, United States; ^6^The Dartmouth Institute for Health Policy and Clinical Practice, Lebanon, NH, United States; ^7^Population Health, Dartmouth Health, Lebanon, NH, United States

**Keywords:** predictive modeling, machine learning, cancer screening, implementation science, breast cancer

## Abstract

**Background:**

This study addresses the critical science challenge of operationalizing social determinants of health (SDoH) in clinical practice. We develop and validate models demonstrating how SDoH predicts mammogram screening behavior within a rural population. Our work provides healthcare systems with an evidence-based framework for translating SDoH data into effective interventions.

**Methods:**

We model the relationship between SDoH and breast cancer screening adherence using data from over 63,000 patients with established primary care relationships within the Dartmouth Health System, an academic health system serving northern New England through seven hospitals and affiliated ambulatory clinics. Our analytical framework integrates multiple machine learning techniques including light gradient boosting machine, random forest, elastic-net logistic regression, Bayesian regression, and decision tree classifier with SDoH questionnaire responses, demographic information, geographic indicators, insurance status, and clinical measures to quantify and characterize the influence of SDoH on mammogram scheduling and attendance.

**Results:**

Our models achieve moderate discriminative performance in predicting screening behaviors, with an average Area Under the Receiver Operating Characteristic Curve (ROC AUC) of 71% for scheduling and 70% for attendance in validation datasets. Key social factors influencing screening behaviors include geographic accessibility measured by the Rural–Urban Commuting Area, neighborhood socioeconomic status captured by the Area Deprivation Index, and healthcare access factors related to clinical sites. Additional influential variables include months since the last mammogram, current age, and the Charlson Comorbidity Score, which intersect with social factors influencing healthcare utilization. By systematically modeling these SDoH and related factors, we identify opportunities for healthcare organizations to transform SDoH data into targeted, facility-level intervention strategies while adapting to payer incentives and addressing screening disparities.

**Conclusion:**

Our model provides healthcare systems with a data-driven approach to understanding and addressing how SDoH shape mammogram screening behaviors, particularly among rural populations. This framework offers valuable guidance for healthcare providers to better understand and improve patients’ screening behaviors through targeted, evidence-based interventions.

## 1 Introduction

The integration of social determinants of health (SDoH) into clinical practice had emerged as a vital frontier in healthcare delivery transformation. Healthcare systems increasingly recognized that addressing SDoH can significantly impact health outcomes and costs (1). Recent evidence demonstrated that higher SDoH needs correlate with greater expenses across both commercial and public insurance systems (1). This recognition highlighted the need for financial incentives for healthcare organizations to incorporate SDoH data into their clinical workflows and decision-making processes (2). Within this evolving landscape, breast cancer screening provided an ideal context for examining SDoH integration, as mammography represented a preventive service with well-documented benefits (3). However, despite being an effective early detection tool for breast cancer, the second leading cause of cancer-related deaths among women globally, mammography screening rates consistently fell below national targets (4). While clinical effectiveness and established guidelines provided strong evidence for mammography benefits, achieving optimal screening rates required addressing complex social, economic, and organizational factors that influenced patient access and engagement (5, 6). This gap between evidence-based recommendations and clinical practice, including delayed scheduling and variations in screening accessibility across healthcare settings, reflects underlying barriers that extend beyond clinical factors alone (7).

Various obstacles to breast cancer screening adherence have been were documented in the literature, including socioeconomic challenges (5, 6), insurance status (5), geographic accessibility (5, 8), transportation limitations (8), cultural beliefs (9, 10), health literacy levels (6), and provider communication effectiveness (9). While these studies provided valuable evidence, they varied in methodological approach from large scale systematic reviews (5) to smaller qualitative investigations (9, 10) with corresponding differences in generalizability and depth of insights. This diversity in methodological approaches across the broader literature made it challenging to develop unified frameworks for understanding how multiple social determinants simultaneously influenced screening behaviors. Collectively, they illustrated how personal, social, and systemic factors could intertwine to create complex patterns of healthcare utilization and screening behaviors (11). Understanding these patterns required recognizing that social determinants do not operate in isolation but rather formed inter-connected networks of influence that shaped individual health decisions.

While the relationships between SDoH and screening behaviors were well-documented, operationalizing SDoH data to improve screening outcomes still presented significant methodological challenges (12–14). The intricate connections between various social determinants and their variable impacts on clinical outcomes demanded sophisticated analytical approaches beyond traditional methods. Qualitative research had provided valuable foundations for identifying the multi-faceted nature of social factors influencing screening behaviors. For example, prior work had explored how economic stability and healthcare access barriers shaped lung cancer screening decisions among Latino communities (15), how health system organizational factors created barriers to implementing social needs screening in primary care settings (16), and how geographic and socioeconomic factors influenced cancer care trajectories and access to treatment (17). However, these qualitative studies are inherently limited in their ability to analyze complex interactions among these factors at scale. Qualitative approaches, while providing rich contextual insights, typically examined small sample sizes that limit statistical power for detecting interaction effects between multiple social determinants. Additionally, the context-specific nature of qualitative research findings often limited their transferability across different healthcare settings and patient populations, making it difficult to establish generalizable relationships between social factors and screening behaviors.

These limitations underscored the need for analytical approaches that can handle large datasets and complex variable interactions. Machine learning approaches offered promising solutions to this complexity, enabling healthcare systems to analyze patterns within SDoH data and develop targeted interventions. These analytical techniques could identify subtle relationships across multiple social determinants simultaneously, which helped to reveal insights that might remain obscured using conventional methods. When healthcare systems could identify which combinations of social factors most strongly predict screening barriers, they could more effectively allocate resources and tailor interventions to the patients who would benefit most. By applying machine learning to SDoH data in the context of breast cancer screening, healthcare organizations could potentially develop personalized approaches to improving screening rates and meet their adherence targets.

In striving toward operationalizing SDoH data and overcoming the limitations of traditional analytical approaches, our study presented an integrated approach to predicting breast cancer screening behaviors. We first developed a generalizable framework for modeling the relationships between social determinants and mammogram scheduling and attendance, providing a structured approach to quantifying these complex influences. We then applied machine learning techniques to transform SDoH data into actionable insights that healthcare systems could use to improve mammogram adherence rates. Through this integrated approach, we aimed to create an evidence-informed methodology for leveraging SDoH data to enhance breast cancer screening outcomes while providing a replicable model that organizations could adapt for other preventive services and health outcomes. This work contributed to the implementation science pipeline, the process of moving research discoveries into routine healthcare practice (18), by providing healthcare systems with quantitative tools to systematically translate SDoH data into actionable screening interventions.

## 2 Materials and methods

### 2.1 General framework for SDoH analysis in mammogram screening behavior

Our generalizable framework included the following steps: data pre-processing and variable construction, model selection and implementation, performance evaluation, and model explainability analysis. We detailed these steps and presented their execution for predicting the probability of mammogram screening behaviors, including both scheduling and attendance.

The comprehensive methodological detailed for each framework component, including specific algorithms, parameter settings, and validation procedures, were provided in Supplementary Methods M1–M4. While this detailed framework was designed for broader application across healthcare systems, we demonstrated its implementation through a specific case study within the Dartmouth Health System.

### 2.2 Case study: the Dartmouth health system

While the framework was designed to be generalizable across different healthcare systems, we applied it specifically to the Dartmouth Health System to demonstrate its practical utility and effectiveness in a real-world setting. Dartmouth Health was an academic health system serving patients across northern New England and nearby communities through seven community hospitals, affiliated ambulatory clinics, and the academic facility Dartmouth Hitchcock Medical Center (19). The system encompassed facilities across Vermont and New Hampshire and utilized an integrated Epic electronic health record (EHR) system that enabled standardized data collection across most clinical sites (19). With over 16,000 employees including 2,300 providers, the system delivered approximately 3 million outpatient visits annually and was recognized as a nationwide leader in rural health (19). This application allowed us to assess the framework’s ability to generate actionable insights within a defined healthcare context before broader implementation in diverse healthcare environments.

#### 2.2.1 Framework overview for operationalizing SDoH

**Figure 1:**
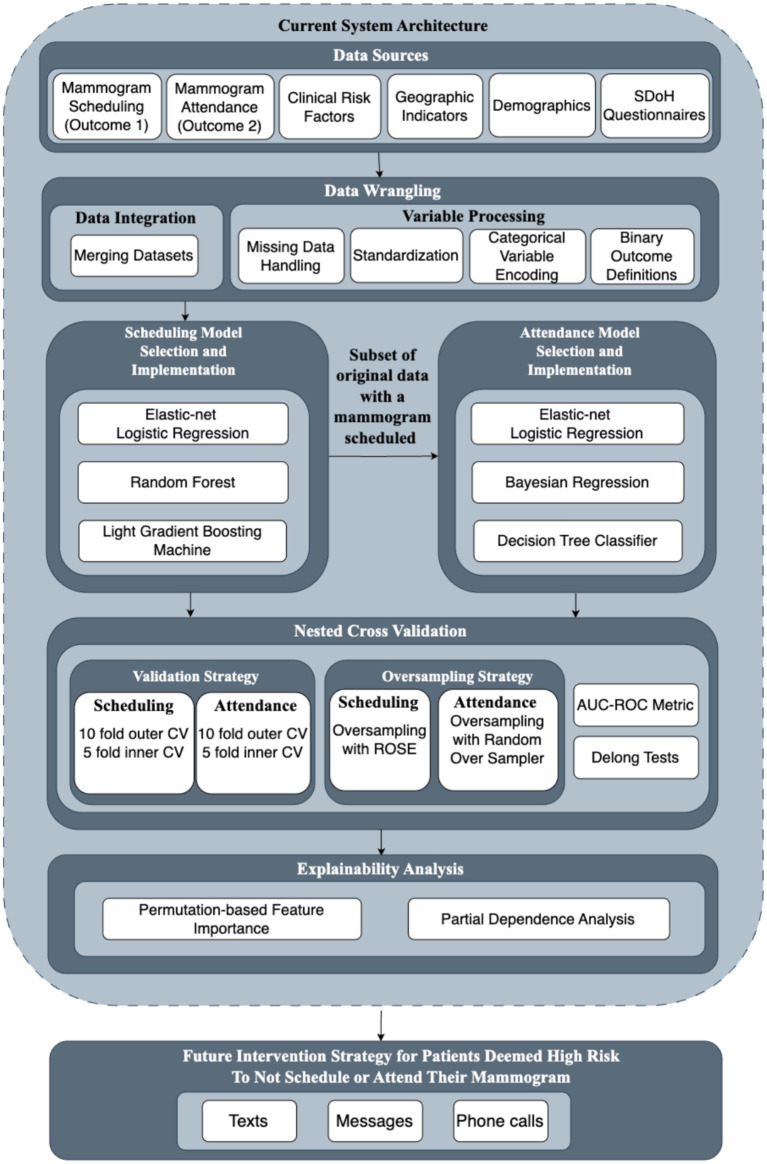
Framework overview for operationalizing SDoH in the Dartmouth health system.

Figure 1 provided a visual representation of our analytical framework for operationalizing SDoH in breast cancer screening programs. This framework, as detailed in Section 2.1, offered a structured approach to integrating diverse healthcare data sources, implementing appropriate machine learning models, validating predictive performance, generating explainable insights, and translating findings into future intervention strategies. Building upon the methodological foundation established by previous work on breast cancer screening prediction models (20), we compared the performance of multiple machine learning techniques. These techniques provided analytical strengths while maintaining interpretability for healthcare practitioners.

#### 2.2.2 Study design and data sources

Our study built upon data from the Dartmouth Health Cancer Screening Outreach Program to develop a framework for operationalizing SDoH into breast cancer scheduling practice. This program operated within Dartmouth Health’s network, which spanned primary and specialty care services throughout New Hampshire and Vermont. A notable characteristic of the Dartmouth Health dataset was its relatively limited racial and ethnic diversity, reflecting the demographic composition of northern New England. This relative homogeneity created a more controlled environment for analyzing other social determinants affecting predominantly rural populations, though it might restrict the model’s ability to capture certain disparities related to race and ethnicity.

The study integrated three primary data sources: (1) patient information from individuals with established primary care relationships (*n* = 63,537), defined as those who received their usual primary care with Dartmouth Health and had had at least one visit with a Dartmouth Health primary care provider in the previous 3 years (medically-homed patients); (2) SDoH questionnaire responses (*n* = 18,359) capturing various dimensions of patient health related social needs; and (3) clinical risk assessment scores from the Epic electronic health record systems, including the Charlson Comorbidity Index (21) and General Adult Risk Scores (22), which provided standardized measures of patient health status and comorbidities.

For demographic variables from the patient information data source, we consolidated 66 language preferences into two groups (English and Others) given that English represented 98% of the population. The original dataset contained eight racial categories (White, American Indian, Asian, Black or African American, Choose not to Disclose, Multi-Racial, Native Hawaiian/Other Pacific Islander, and Unknown). Due to small sample sizes in several subcategories, we consolidated these into five major groups: White, Asian, Black, Hispanic, and Other.

SDoH data were collected using Dartmouth Health’s standardized Adult Screener questionnaire embedded in Epic. Patients’ self-reported answers were captured through the screener administered via the MyDH patient portal or during clinical visits. The screening tool assessed 37 social determinant domains including housing stability, food security, transportation access, social isolation, financial strain, employment status, and healthcare access barriers (Supplementary Tables S3, S5). While Epic’s SDoH screening modules had demonstrated implementation feasibility in clinical settings (23), formal psychometric validation data for the complete screening instrument had not been published. Our primary outcome measure was mammogram scheduling status and attendance status, which served as our indicators of patient engagement with breast cancer screening.

#### 2.2.3 Study population and inclusion criteria

The study population encompassed female patients aged 50–75 years receiving active care at Dartmouth Health primary care clinics, with active care defined as having completed a primary care visit at the health system within the previous 3 years. To maintain focus on adherence to standard Dartmouth Health breast cancer screening schedules for women with average risk, we excluded patients with breast cancer history or elevated risk factors that would necessitate different scheduling protocols. To ensure consistent screening practices across study sites, we also excluded two clinical sites that utilized different appointment scheduling protocols from the standard Dartmouth Health approach. While these sites demonstrated higher adherence rates due to automatic scheduling, their inclusion would have confounded our analysis of standard care patterns by introducing scheduling protocol variability.

#### 2.2.4 Model validation

To ensure the external validity of our findings, we employed our models on a hold-out test set (20% of the data) that was not used during model development or hyperparameter tuning. This approach provided an unbiased assessment of model generalizability to new patients within the Dartmouth Health System. We applied consistent performance evaluation metrics between our development and test phases, allowing us to directly compare predictive capabilities and quantify how effectively our models can identify screening patterns in previously unseen data. This evaluation on independent data helped determine whether the relationships identified during model training remained stable when applied in new contexts, providing healthcare systems with confidence that the implementation insights generated by our models would be reliable and actionable in clinical settings.

### 2.3 Sensitivity analyses and secondary analyses

To assess the robustness of our findings to different analytical assumptions, we conducted sensitivity analyses focusing on missing data handling approaches. Specifically, we performed complete case analyses using only patients with complete SDoH questionnaire data as sensitivity checks for our primary imputation-based approach. These analyses used identical modeling frameworks and performance evaluation metrics as described in Section 2.1 to ensure comparability with our primary results.

Additionally, we conducted comprehensive secondary analyses to provide deeper insights into factors influencing mammogram screening behaviors, including age-stratified evaluations, SDoH-only models, patient-level models, and clinic-level analyses. Detailed methodologies and results for all secondary analyses are presented in Supplementary materials S1.1–S1.5 (Scheduling analyses) and Supplementary materials S2.1–S2.5 (Attendance analyses).

## 3 Results

### 3.1 Data structure and missingness

Our analysis of SDoH questionnaire data revealed substantial variation in response completeness across the 37 administered questions. Missingness rates ranged from 10.2 to 92.4%, with a median missingness of 73.8% across all questions (Supplementary Table S1). This evaluation identified 11 questions that exceeded our pre-established 80% missingness threshold, which were subsequently excluded from model development to ensure implementation reliability. The excluded questions primarily addressed sensitive domains such as mental health status, substance use behaviors, and detailed information regarding past scheduling experiences.

Examination of the dataset revealed distinct patterns in both scheduling and attendance behaviors. Scheduling rates, calculated as the proportion of all eligible women aged 50–75 who had a mammogram scheduled, showed substantial variation across clinical sites (4.4–21.3%), insurance types (Medicare: 16%; Commercial: 12.9%), age groups, and neighborhood deprivation levels. For attendance, missed appointment rates varied by clinical site (1.9–9.1%), insurance status (Medicaid Managed: 13%; Blue Cross: 4%), and racial demographics (Asian: 0.8%; Hispanic: 8.1%) (Supplementary Table S4). We found a linear relationship between neighborhood deprivation and missed appointments (ADI 1: 1.6%; ADI 10: 11.4%) and higher attendance in urban areas compared to rural settings. SDoH questionnaire responses indicated that housing instability (multi-residence: 12.4% vs. single-residence: 4.8% missed appointments), transportation barriers (unable to work due to transportation: 18.8% vs. no barriers: 4.9%), food insecurity (often: 14.3% vs. never: 4.9%), and health literacy challenges were associated with lower scheduling rates and higher missed appointment rates (Supplementary Tables S2, S3, S5).

### 3.2 Analytical framework performance

#### 3.2.1 Scheduling model performance

The light gradient boosting model demonstrated a moderate average out-of-sample performance in our cross-validation scheme (AUC = 0.709), followed by random forest (AUC = 0.702) and elastic-net logistic regression (AUC = 0.608). Both tree-based models significantly outperformed the logistic regression approach, with the light gradient boosting model showing a statistical advantage over logistic regression (AUC difference = 0.050, *p* < 0.001) and random forest similarly demonstrating superior performance compared to logistic regression (AUC difference = 0.05, *p* < 0.001). The difference between gradient boosting and random forest models was minimal (AUC difference = 0.0001) and not statistically significant (*p* = 0.972), confirming that both tree-based approaches had comparable predictive power for this scheduling behavior prediction.

The gradient boosting model, our best-performing approach, showed strong consistency across validation scenarios. The model’s performance ranged from 0.707 (worst AUC on validation sets) to 0.711 (best AUC on validation sets), indicating stable predictive performance. Our AUC on the held-out test set, which predicts model performance on unseen data, also achieved a relatively similar AUC of 0.67. This stability was particularly important for healthcare systems implementing SDOH-informed scheduling programs across diverse communities.

#### 3.2.2 Attendance model performance

For attendance prediction, we used an identical approach to compare three machine learning models: Bayesian regression (AUC = 0.702), elastic-net logistic regression (AUC = 0.699), and decision tree classifier (AUC = 0.666).

Delong’s test showed that these three models performed comparably (AUC difference Bayes-Log: 0.004, *p* > 0.05) (AUC difference bayes-tree: 0.00423, *p* > 0.05) (AUC difference log-tree: 0.00323, *p* > 0.05). Given this comparable performance, we selected logistic regression as our final model for its computational simplicity and independence from prior assumptions. This selected model demonstrated moderate consistency across validation datasets. Performance ranged from AUC = 0.6531 to AUC = 0.7851, with an average validation AUC of 0.7282. When evaluated on the held-out test set, the model maintained robust performance (AUC = 0.699), which showed somewhat consistent predictive power.

#### 3.2.3 Permutation-based variable importance

**Figure 2:**
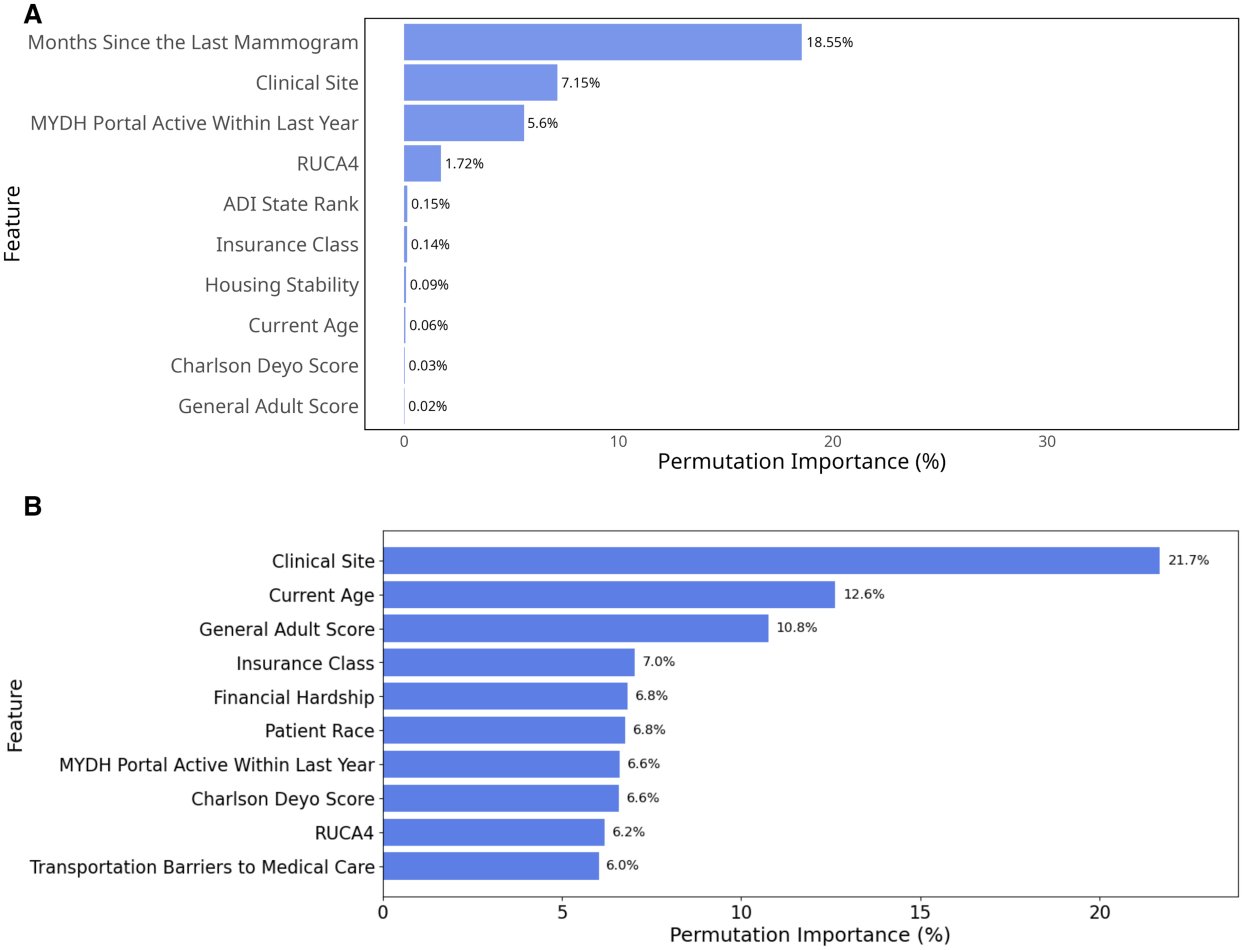
Permutation importance based on percentage decrease in AUC. **(A)** Top 10 most important variables in the scheduling model; **(B)** Top 10 most important variables in the attendance model.

Our variable importance analysis from light gradient boosting machine (scheduling model) and elastic-net logistic regression (attendance model) using a permutation-based approach identified key social drivers for future implementation focus (Figure 2).

For the scheduling model (Figure 2), months since the last mammogram emerged as the strongest individual predictor with a permutation importance value of 18.55%. Clinical site was the second most influential factor (7.15%), followed by MYDH Portal active within last year (5.6%). When considering cumulative effects, these top three features together represented more than 30% of the total permutation importance, suggesting that temporal, demographic, and organizational factors were particularly crucial for non-scheduling behavior. Geographic and socioeconomic factors also showed some influence, with RUCA4 (1.72%) and ADI state rank (0.15%) completing the top five predictors. Almost all traditional SDoH questionnaire responses such as homelessness (0.01%), food insecurity (0.01%), and financial hardship (0.01%) showed limited predictive power in our model and were therefore excluded from the diagram. This less prominent role of direct SDoH questionnaire measures compared to geographic and facility-level indicators suggested that social determinants might exert their influence through complex pathways that are better captured by community-level metrics and healthcare delivery characteristics than by individual self-reported social needs.

For the attendance model, clinical site was the most influential variable, contributing 21.7% to model performance, followed by current age (12.6%) and General Adult Risk Score (10.8%). This importance indicated that both site-level factors and patient health burden strongly influence attendance (Figure 2B). Insurance class (7%), Financial hardship (6.8%) and patient race (6.8%) also played notable roles, suggesting that insurance coverage, economic constraints, and demographic factors affected screening adherence. In contrast to the scheduling model, the attendance model excluded months since the last mammogram (the strongest scheduling predictor) to avoid data leakage, as temporal information was incorporated into the attendance outcome definition (see Methods 2.1.1).

#### 3.2.4 Partial dependence plots

**Figure 3:**
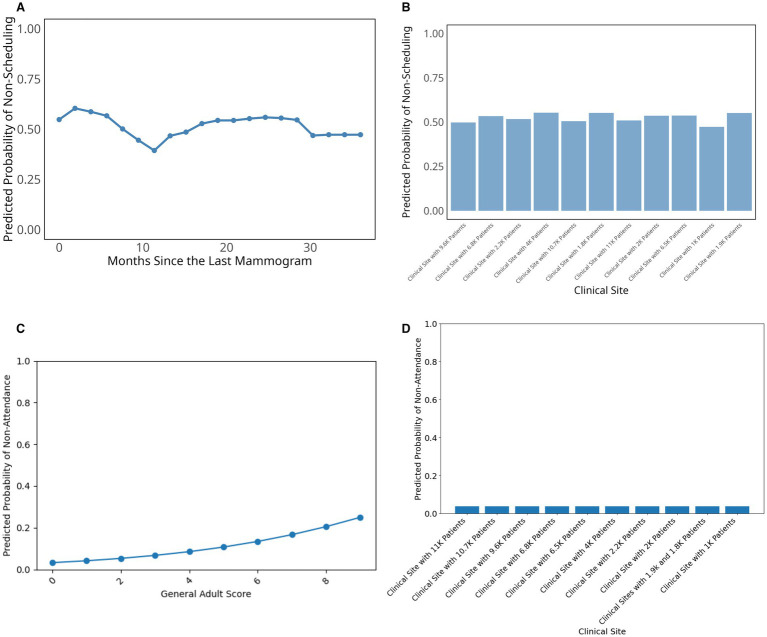
Partial dependence plots for top predictors in breast cancer scheduling and attendance prediction. **(A)** Top numerical variable in the scheduling model; **(B)** Top categorical variable in the scheduling model; **(C)** Top numerical variable in the attendance model; **(D)** Top categorical variable in the attendance model.

To further examine how key social drivers influence breast cancer screening behavior, we plotted partial dependence plots for the most influential predictors (Figure 3). For the scheduling model, months since the last mammogram showed a distinct temporal pattern with particularly higher probability of not scheduling within the first few months, followed by a significant drop around 10–12 months, and subsequent fluctuations that stabilize after approximately 30 months (Figure 3A). Among categorical predictors, clinical site demonstrated some variation in the probability of not scheduling across different healthcare facilities, with relatively consistent predicted probabilities ranging between approximately 0.45 and 0.55 (Figure 3B).

For the attendance model (Figures 3C,D), the General Adult Risk Score showed a clear positive relationship with non-attendance probability, with higher scores indicating greater health complexity and comorbidity burden. These higher scores were associated with increased likelihood of missing scheduled appointments, rising from near-zero probability at low scores to approximately 0.25 at the highest health complexity scores (Figure 3C). In contrast, clinical site showed minimal variation in attendance patterns, with predicted non-attendance probabilities remaining consistently low (below 0.1) across most healthcare facilities (Figure 3D). While clinical site was one of the top important variables in our variable importance analysis, the practical differences in attendance rates between sites were modest once patient-level factors are accounted for.

Additional variables examined in our analysis, including Charlson Comorbidity Index, housing stability, patient race, age, and various social determinants of health measures showed relatively minimal impact on mammography screening or little variation in not scheduling (Supplementary Figure S1) and screening non-attendance probability (Supplementary Figure S2).

## 4 Discussion

Our study showed that machine learning approaches can effectively identify the factors that influence breast cancer scheduling and attendance behavior within a healthcare system. While we initially examined SDoH as potential drivers of screening patterns, our findings revealed that healthcare systems might achieve better impact by focusing on factors within their direct control. The light gradient boosting model achieved clinically meaningful performance comparable to other predictive models addressing SDoH-related scheduling outcomes (24, 25). Furthermore, the elastic-net logistic regression model achieved modest performance relative to other predictive models when addressing SDoH related attendance outcomes (26, 27). Our findings highlighted several key implementation domains: temporal patterns in scheduling behavior revealed the dynamic nature of patient engagement; facility-level variables emerged as important predictors, reflecting the influence of organizational characteristics; and social determinants and geographic factors demonstrated the impact of community context, though to a lesser degree than anticipated. While the model’s performance reflected the inherent challenges of quantifying social factors, it might provide healthcare systems with actionable insights for implementing scheduling programs that address both organizational and community-level barriers while accounting for individual patient characteristics.

### 4.1 Implementation implications

Our analysis revealed that the relationship between social determinants and screening behavior involved multiple interacting factors. The light gradient boosting model’s ability to capture these relationships (AUC = 0.709), together with similar performance from the random forest approach (AUC = 0.702), suggested that accounting for non-linear interactions between social drivers might help healthcare systems better understand scheduling behavior patterns.

Consistent with established literature, our models confirmed that geographic accessibility (RUCA) and socioeconomic factors (ADI state rank) influence screening behaviors (5, 8). However, our variable importance analysis revealed a hierarchy of influence that differs from traditional approaches focused primarily on individual-level social barriers. Temporal factors (months since last mammogram) and organizational factors (clinical site) emerged as the strongest predictors, suggesting that healthcare systems might achieve more immediate impact through system-level interventions rather than attempting to address individual patients’ social circumstances. This finding highlighted a different priority than much of the existing mammography literature, which emphasized individual-level barriers such as transportation, cultural beliefs, and health literacy (6, 9, 10). While these individual SDoH factors remained important in our descriptive analyses, our machine learning approach revealed that facility-level variations and care patterns were more predictive of scheduling and attendance behavior. This pattern was particularly true for traditional SDoH questionnaire response, where individual measures such as homelessness, food insecurity, and financial hardship each contributed less than 0.01% to variable importance. This minimal predictive power might have reflected the substantial missingness in SDoH data, potential underreporting of sensitive information in clinical settings, or that geographic and organizational indicators served as more reliable proxies for underlying social determinants.

For Dartmouth Health specifically, this suggested that standardizing practices across clinical sites might yield greater improvements in screening rates than traditional patient-education or transportation-assistance programs. For attendance behavior, the evidence suggested a dual approach combining system-level standardization with targeted interventions. This recommendation was supported by our clinic-level analysis for scheduling (Supplementary material S1.4), which confirmed substantial performance variation across sites. However, the clinic-level analysis for attendance (Supplementary material S2.4) showed inconsistent results, limiting conclusions about organizational effects.

Our findings also illuminated the complex interplay between organizational and social factors that traditional regression approaches often missed (11). The elastic-net logistic regression model’s performance in capturing attendance patterns (AUC = 0.698) demonstrated that patient health complexity (General Adult Risk Score) and digital engagement (portal activity) were critical factors that complement traditional socioeconomic predictors. This insight provided healthcare systems with a more nuanced understanding of how to target interventions across different patient populations.

Beyond these specific findings for breast cancer screening, our systematic framework laid the groundwork for analyzing SDoH’s influence on other preventive health behaviors, demonstrating the potential for broader applications in improving routine preventive care utilization. The methodological approach of combining individual-level social determinants with organizational and temporal factors could be adapted to examine colorectal cancer screening, cervical cancer screening, and other preventive services where similar complex interactions between social drivers and healthcare delivery factors likely influence patient engagement.

### 4.2 Methodological contributions

Our analytical approach offered several methodological contributions to healthcare delivery and the implementation science. First, we demonstrated a novel approach to operationalizing SDoH in breast cancer scheduling and attendance practices, providing healthcare systems with a framework to translate social determinant screening tools into actionable screening strategies. Unlike previous work analyzing nationwide census tract-level scheduling rates and focused primarily on geographic accessibility and demographics, our study examined individual-level data integrating clinical, behavioral, and social determinants within a healthcare system context (28). This focus allowed us to identify specific patient-level factors that directly influence scheduling decisions, rather than ecological correlations at the population level.

Second, our modeling framework effectively functioned as a poly-social risk score system, aggregating multiple social determinants to quantify their combined influence on screening adherence. This approach moved beyond examining isolated social determinants to consider how they collectively impact health behaviors. Third, the age-stratified analysis (Supplementary material S1.2) revealed important variations in these poly-social risk profiles across demographic groups, suggesting the need for age-specific implementation strategies that account for different SDoH impacts across the lifespan. Finally, our application of light gradient boosting models and elastic-net logistic regression models and partial dependence plots revealed important non-linear patterns in the relationship between months since the last mammogram and non-scheduling probability—a critical insight that traditional regression approaches would likely miss. For the attendance model, our elastic-net logistic regression approach similarly captured complex relationships between organizational factors, patient characteristics, and social determinants, though with different key predictors than the scheduling model. These findings demonstrated the value of machine learning approaches in capturing complex relationships between certain social determinants and screening behavior.

The development of a unified modeling framework that incorporated both individual-level social drivers and system-level factors provided healthcare organizations with a template for analyzing their own screening programs. This approach could be particularly valuable as healthcare systems work to improve cancer screening rates for their medically-homed populations while effectively integrating SDoH data into their quality improvement initiatives.

### 4.3 Future directions in implementation science

The substantial missingness in our SDoH questionnaire data, with a median of 73.8% across questions, reflected common implementation challenges in clinical settings. Our analysis excluded 11 questions that exceeded the 80% missingness threshold, which primarily addressed sensitive domains such as mental health status, substance use behaviors, and detailed information regarding past scheduling experiences. Health literacy, a factor that prior research has demonstrated to influence mammography screening adherence (6), represented another domain affected by substantial missingness, restricting our ability to comprehensively assess its influence on patient screening decisions. These exclusions represented a methodological consideration because these sensitive domains might be critical drivers of patient decision-making regarding mammogram scheduling and attendance. More complete data on these domains could potentially alter our understanding of the factors driving screening behavior within healthcare systems. These data collection challenges highlighted the need for alternative approaches to capture important behavioral determinants. However, our framework was designed to be adaptable and can incorporate these variables when improved collection methods make such data available in future implementations.

These implementation challenges underscored the importance of systematic approaches to translating our findings into practice. The Consolidated Framework for Implementation Research (CFIR) offered a valuable lens for future efforts to translate our findings into practice. Though our current work focused on quantitative modeling rather than a full CFIR implementation, our findings provided a foundation for subsequent mixed-methods approaches that could more fully leverage implementation science frameworks.

For example, the facility-level variations identified in our model aligned with CFIR’s ‘inner setting’ domain, suggesting that organizational culture and readiness for implementation played important roles in both scheduling and attendance behaviors. Future work could build on our quantitative findings by using qualitative methods to explore how these organizational factors influenced practices and how interventions might be tailored to different clinical settings. Similarly, our findings related to geographic and socioeconomic factors corresponded to CFIR’s ‘outer setting’ domain, highlighting the importance of understanding patient needs and resources within their communities. Further investigations could provide deeper insights into how these community factors shaped decisions and how healthcare systems might better address them.

As healthcare systems consider implementing SDoH-informed interventions, CFIR and other implementation science frameworks could provide valuable guidance for assessing feasibility, sustainability, and potential barriers. Our work represented an important first step in this direction by providing quantitative evidence of key relationships that future implementation efforts should consider.

### 4.4 Future research priorities

Our model was developed and validated within the Dartmouth Health system, which served a population with limited racial, ethnic, and linguistic diversity. This demographic homogeneity might have limited our ability to capture important language-related barriers to screening access and communication, and might have restricted the generalizability of our findings to more diverse healthcare settings and populations. Although our findings indicated small racial differences, future work should validate these approaches in healthcare systems serving more diverse communities to ensure broader applicability. It was important to note, however, that we had designed our work as a generalizable framework that could perform well in other situations and could incorporate more diverse racial groups and other demographic aspects if the necessary data were available.

Moreover, our modeling approach assumed that the ratio between screening and non-screening populations would remain stable over time. This assumption might not have held in different implementation contexts or as scheduling programs evolve. Healthcare systems implementing similar approaches should carefully consider their local population characteristics and mammogram scheduling patterns. Additionally, while our model demonstrated modest predictive performance within our system, its generalizability to other healthcare settings might be limited by differences in organizational structure, population characteristics, screening protocols, and the substantial missingness in our SDoH data, which might have limited our ability to fully capture social determinant influences. Future research should explore how these models could be adapted and calibrated for different healthcare contexts.

Beyond expanding the population and removing assumptions, we identified several priority areas for future research. First, the development of dynamic modeling approaches that could adapt to changing population characteristics and scheduling patterns would enhance the robustness of our framework. Additionally, integrating SDoH-informed scheduling models with other preventive care programs could create more comprehensive implementation strategies. Investigation of facility-level variations in scheduling patterns would have further identified best practices for implementation. Finally, extending our analytical framework to other scheduling programs, such as colorectal and cervical cancer scheduling, would increase the broader applicability of SDoH-informed modeling approaches and strengthen the overall impact of our generalizable framework across diverse healthcare settings.

### 4.5 Conclusion

Our study provided healthcare systems with a data-driven approach to understanding and addressing how social determinants shape breast cancer scheduling practices. Our findings suggested that machine learning approaches could help healthcare systems develop more effective, targeted implementation strategies. As healthcare systems worked to meet cancer screening targets for their medically-homed populations, approaches that systematically analyzed and addressed social determinants of health could have become increasingly valuable for improving adherence and reducing disparities.

For the scientific community, these findings offered two primary contributions. First, our results demonstrated the relative influence of different predictors on screening behaviors, highlighting that healthcare systems might achieve greater impact by focusing on factors within their direct control rather than attempting to address individual patients’ social circumstances alone. Second, our framework enabled identification of patients at highest risk of not scheduling or attending appointments, providing a practical tool for targeted intervention strategies.

Looking ahead, our quantitative findings provided a foundation for future implementation science approaches that could more fully leverage frameworks like CFIR to translate these insights into practice. By combining machine learning approaches with implementation science, healthcare systems could develop more comprehensive strategies for addressing the complex interplay between social determinants and screening behaviors, ultimately improving health outcomes for diverse patient populations. Healthcare systems and researchers could adapt this approach using their own data to develop targeted interventions and improve mammography adherence within their specific patient populations and organizational contexts. Through such systematic approaches to understanding and addressing screening behaviors, healthcare system could potentially work toward more effective, evidence-based strategies for reducing disparities and improving preventive care delivery.

## Data Availability

The data analyzed in this study is subject to the following licenses/restrictions: the dataset analyzed in this study contain de-identified patient health information from the Dartmouth Health System and cannot be made publicly available due to privacy restrictions. Data access would require formal agreements with Dartmouth Health and IRB approval. Requests to access these datasets should be directed to Wesley J. Marrero, wesley.marrero@dartmouth.edu.
