# Supplementary material for: Modeling the impact of social determinants on breast cancer screening: a data-driven approach
Source: Front Med (Lausanne). 2025 Aug 20;12:1644287. doi: 10.3389/fmed.2025.1644287 (PMC12405414; doi:10.3389/fmed.2025.1644287)
Supplement: Supplementary file 1 [file Data_Sheet_1.pdf]

SUPPLEMENTARY FILES

Supplementary Figures

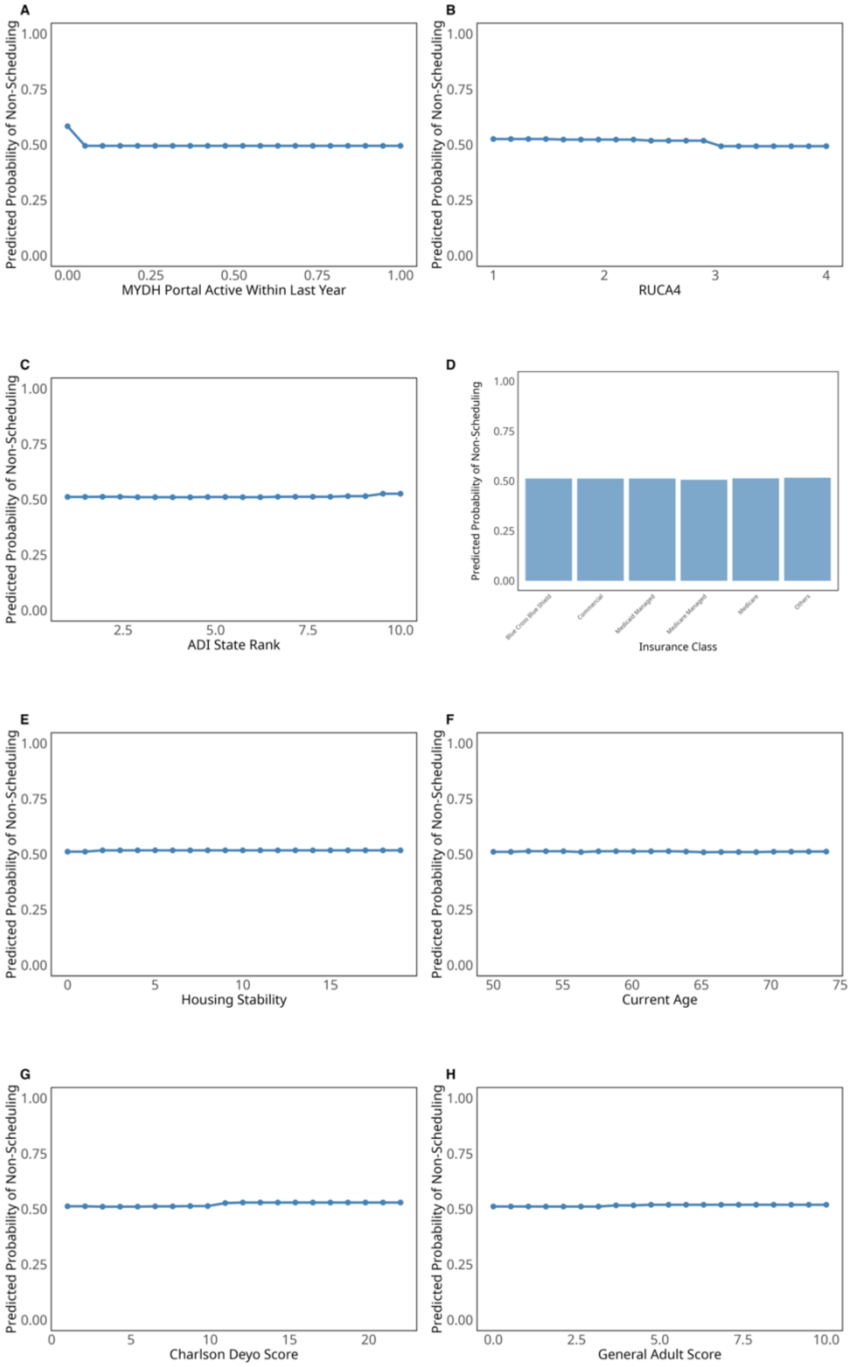

Supplementary Figure S1: Partial Dependence Plots for Additional Variables. The plots display the relationship between various factors and mammography scheduling probability: (A) MYDH Portal Active Within Last Year, (B) RUCA-4, (C) ADI State Rank; (D) Insurance Class; (E) Housing Stability; (F) Current Age; (G) Charlson Deyo Score; (H) General Adult Score. Most of these variables show minimal influence on scheduling probability, with relatively flat relationships compared to the stronger predictors shown in Figure 3 and 4.

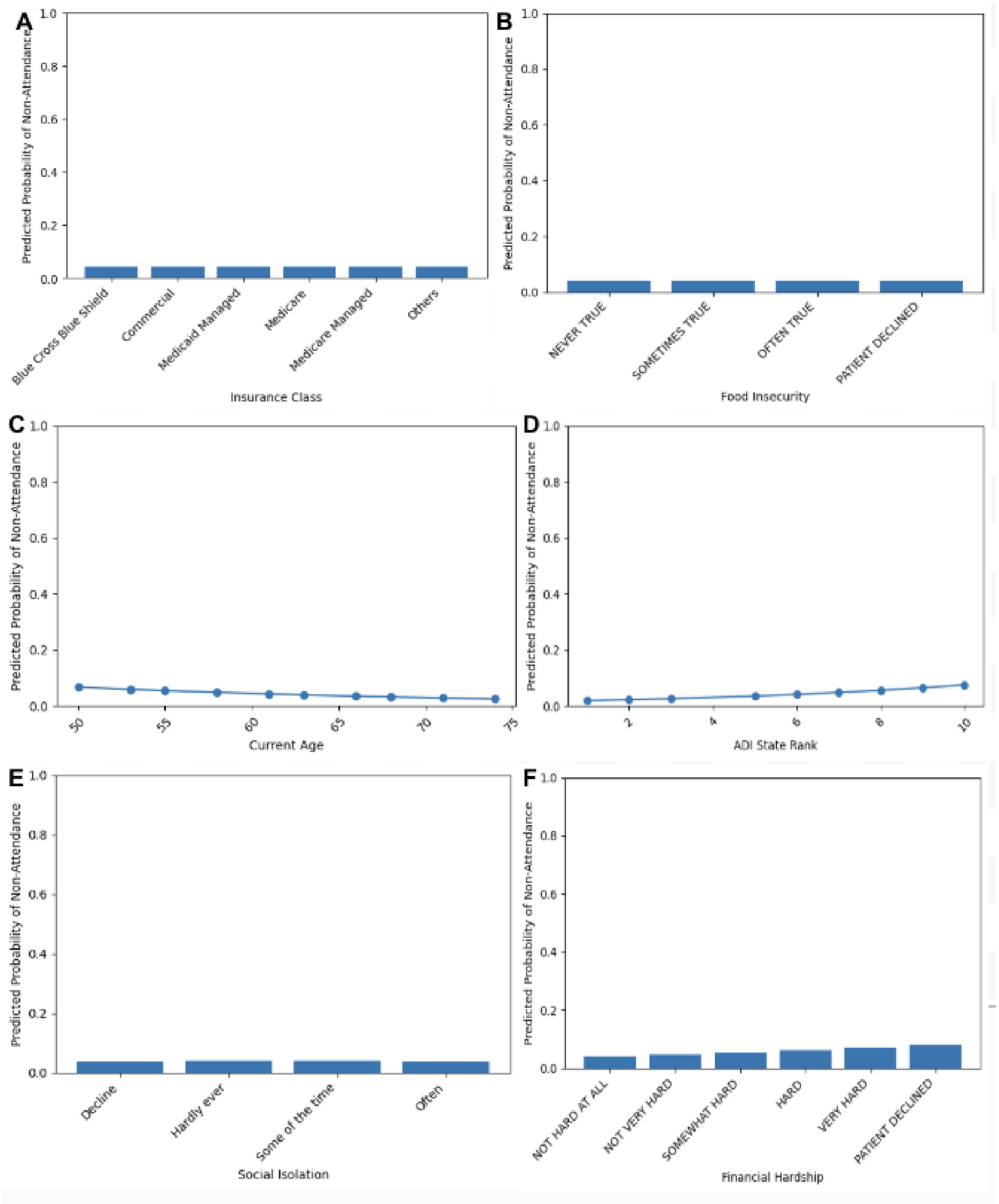

Supplementary Figure S2: Partial Dependence Plots for Additional Variables. The plots display the relationship between various factors and mammography scheduling probability: (A) MYDH Portal Active Within Last Year, (B) RUCA-4, (C) Current Age, (D) Patient Race, (E)Insurance Class, (F) Financial Hardship.

Supplementary Tables

*Supplementary Table S1: Missingness Per Question. Missing Percentage Per Question (%) is calculated as (Missing Responses/Total Eligible Patients)*100. Missing responses include both patients who never encounter the question and those who encountered it but did not provide a valid response (excluding 'Patient Refused' and 'Declined' which were retained as meaningful responses.*

QUESTION	Number of Responses	Total Responses	Evaluable Responses	Missing Percentage Per Question (%)
How hard is it for you to pay for the very basics like food, housing, medical care, and heating?	18357	18583	226	1.22
Within the past 12 months, you worried that your food would run out before you got the money to buy more.	18313	18548	235	1.27
In the last 12 months, was there a time when you were not able to pay the mortgage or rent on time?	18277	18568	291	1.57
In the past 12 months, has lack of transportation kept you from meetings, work, or from getting things needed for daily living?	18145	18709	564	3.01
In the past 12 months, has lack of transportation kept you from medical appointments or from getting medications?	18174	18801	627	3.33
How often do you feel isolated from others?	18183	18883	700	3.71
In the last 12 months, was there a time when you did not have a steady place to sleep or slept in a shelter (including now)?	17761	18633	872	4.68
Within the past 12 months, the food you bought just didn't last and you didn't have money to get more.	18295	19216	921	4.79
How often do you need to have someone help you when you read instructions, pamphlets, or other written material from your doctor or pharmacy?	17681	18667	986	5.28
In the last 12 months, how many places have you lived?	17787	19819	2032	10.25
In the past 12 months has the electric, gas, oil, or water company threatened to shut off services in your home?	9575	18517	8942	48.29
How often do you feel that you lack companionship?	3528	18486	14958	80.92
How often do you feel left out?	3524	18476	14952	80.93
What was your main activity during most of the last 12 months?	3509	18442	14933	80.97
Do you have any legal issues that you are having difficulty resolving, such as disability, custody, parole, eviction, restraining orders, or other?	3507	18450	14943	80.99
Do you feel unsafe in your relationships at home, school or work?	3502	18444	14942	81.01
Has someone threatened to hurt you, your family or your pets?	3499	18449	14950	81.03
Have you ever felt that someone wants to hurt you?	3495	18438	14943	81.04
Does anyone try to keep you from having contact with others or doing things outside your home?	3495	18441	14946	81.05
Do you have reliable use of a phone?	3483	18424	14941	81.1

**Do you have reliable access to the internet?**	3480	18427	14947	81.11
**Social Isolation Score**	3484	18645	15161	81.31
**Are you able to speak on the phone in a safe and private manner?**	3403	18446	15043	81.55
**Are you able to access the internet in a safe and private manner?**	3336	18444	15108	81.91
**Within the last year, have you been humiliated or emotionally abused in other ways by your partner or ex-partner?**	1878	18377	16499	89.78
**Within the last year, have you been afraid of your partner or ex-partner?**	1874	18379	16505	89.8
**Within the last year, have you been raped or forced to have any kind of sexual activity by your partner or ex-partner?**	1872	18379	16507	89.81
**Within the last year, have you been kicked, hit, slapped, or otherwise physically hurt by your partner or ex-partner?**	1870	18379	16509	89.83
**What do you have trouble paying for?**	654	18711	18057	96.5
**At any time in the past 12 months, were you homeless or living in a shelter (including now)?**	476	18364	17888	97.41
**How often do you need to have someone help you when you read instructions, pamphlets, or other written material from your doctor or pharmacy?**	474	18365	17891	97.42
**In the past 12 months, how many times have you moved where you were living?**	476	18470	17994	97.42
**How hard is it for you to pay for the very basics like food, housing, medical care, and heating?**	1	18359	18358	99.99
**In the past 12 months, has lack of transportation kept you from medical appointments or from getting medications?**	1	18359	18358	99.99
**In the past 12 months, has lack of transportation kept you from meetings, work, or getting things needed for daily living?**	1	18359	18358	99.99
**Within the past 12 months, the food you bought just didn't last and you didn't have money to get more.**	1	18359	18358	99.99
**Within the past 12 months, you worried that your food would run out before you got money to buy more.**	1	18359	18358	99.99

Supplementary Table S2: Consolidated Demographic Counts

Category	Number Not Scheduled	Number Scheduled	Proportion Not Scheduled
**Clinical Site**			
Clinical Site with 11K Patients	1878	304	86%
Clinical Site with 10.7K Patients	537	130	80%
Clinical Site with 9.6K Patients	804	212	79%
Clinical Site with 6.8K Patients	855	109	89%
Clinical Site with 6.5K Patients	964	145	87%
Clinical Site with 4K Patients	240	21	92%

Clinical Site with 2.2K Patients	192	38	83%
Clinical Site with 2K Patients	273	34	89%
Clinical Site with 1.9K Patients	306	14	96%
Clinical Site with 1.8K Patients	394	27	94%
Clinical Site with 1K Patients	100	27	79%
**Insurance Class**			
Blue Cross Blue Shield	2517	396	86%
Commercial	1742	257	87%
Medicare	949	181	84%
Medicare Managed	866	158	85%
Others	316	40	89%
Medicaid Managed	153	29	84%
**Race**			
White	6021	979	86%
Others	224	32	87%
Asian	136	25	84%
Hispanic	108	19	85%
Black	54	6	90%
**Language**			
English	6490	1050	86%
Others	53	11	83%
**Charlson Deyo Score**			
1	184	33	85%
2	1767	264	87%
3	2028	297	87%
4	1121	197	85%
5	622	139	82%
6	342	53	87%
7	159	24	87%
8	100	24	81%
9	67	11	86%
10	46	6	88%
11	37	4	90%
12	36	6	85%
13	11	1	91%
14	12	1	92%

15	4	0	100%
16	4	0	100%
17	1	0	100%
18	1	0	100%
19	1	1	40%
**General Adult Score**			
0	2540	350	88%
1	2539	435	85%
2	963	189	84%
3	336	65	84%
4	110	18	86%
5	30	4	88%
6	14	0	100%
7	6	0	100%
8	3	0	100%
9	1	0	100%
10	1	0	100%
**MyDH Active Within Last Year**			
Yes	6392	1050	86%
No	151	11	93%
**Age Group**			
50-54	1388	216	87%
55-59	1480	232	86%
60-64	1601	223	88%
65-69	1269	229	85%
70-74	805	161	83%
**State Rank (ADI)**			
1	808	118	87%
2	763	115	87%
3	788	121	87%
4	737	101	88%
5	725	117	86%
6	671	116	85%
7	578	122	83%
9	516	96	84%
8	576	84	87%

10	381	71	84%
**Rural-Urban Commuting Area**			
1	2408	359	87%
2	443	62	88%
3	2355	396	86%
4	1337	244	85%

Supplementary Table S3: Consolidated Question Counts

Category	Number Scheduled	Proportion Scheduled	Number Not Scheduled	Proportion Not Scheduled
**Unable to pay rent or mortgage in the past 12 months**				
No	987	0.1407	6026	0.8592
Yes	42	0.1183	313	0.8814
Patient Declined	16	0.2133	59	0.7844
Patient Refused	16	0.0994	145	0.9001
**Number of residences in the past 12 months**				
0	8	0.1905	34	0.8059
1	986	0.1397	6074	0.8603
2	59	0.1379	369	0.8619
3	6	0.1132	47	0.8849
4	0	0.0	8	1.0
5	0	0.0	2	1.0
7	0	0.0	2	1.0
9	0	0.0	1	1.0
10	0	0.0	2	1.0
12	1	0.5	1	0.4
13	0	0.0	2	1.0
19	1	1.0	0	0.0
33	0	0.0	1	1.0
**Unable to access medication due to transportation difficulties**				
No	1033	0.1397	6363	0.8603
Yes	18	0.1731	86	0.8255
Patient Declined	10	0.0962	94	0.903
**Feels isolated from others**				
Hardly ever	863	0.1419	5219	0.8581
Some of the time	133	0.1329	868	0.867

Often	35	0.1311	232	0.8685
Decline	30	0.1181	224	0.8815
**Difficulty paying for necessities**				
Not Hard at All	721	0.1424	4343	0.8576
Not Very Hard	197	0.1315	1301	0.8684
Somewhat Hard	93	0.1418	563	0.858
Patient Declined	20	0.1149	154	0.8845
Hard	16	0.1345	103	0.8646
Very Hard	14	0.1505	79	0.8481
**Concerns about inadequate access to food in the past 12 months**				
Never True	984	0.1411	5990	0.8589
Sometimes True	44	0.1239	311	0.8758
Patient Declined	20	0.1036	173	0.8959
Often True	13	0.1585	69	0.8398
**Difficult reading medical material**				
Never	965	0.1386	5997	0.8614
Rarely	49	0.1546	268	0.845
Sometimes	21	0.1338	136	0.8655
Decline	11	0.1358	70	0.8628
Always	8	0.1905	34	0.8059
Often	7	0.1556	38	0.8415
**Lacked a consistent residence in the past 12 months**				
No	1044	0.1405	6389	0.8595
Yes	8	0.1026	70	0.8963
Patient Refused	5	0.0769	60	0.922
Patient Declined	4	0.1429	24	0.8528
**Unable to work due to lack of transportation**				
No	1036	0.1401	6357	0.8599
Yes	15	0.1562	81	0.8424
Patient Declined	10	0.087	105	0.9124
**Insufficient access to food in the past 12 months**				
Never True	991	0.1399	6091	0.8601
Sometimes True	40	0.1515	224	0.848
Patient Declined	21	0.1082	173	0.8913

Often True	9	0.1406	55	0.8575

Supplementary Table S4: Consolidated Demographic Counts-- Attendance

Category	Number Not Attended	Number Attended	Proportion Not Attended
**Clinical Site**			
Clinical Site with 11K Patients	62	1192	4.94%
Clinical Site with 10.7K Patients	117	1176	9.05%
Clinical Site with 9.6K Patients	10	1202	0.83%
Clinical site with 6.8K Patients	19	446	4.09%
Clinical Site with 6.5K Patients	26	549	4.52%
Clinical Site with 4K Patients	16	337	4.53%
Clinical Site with 2.2K Patients	19	256	6.91%
Clinical Site with 2K Patients	8	125	6.02%
Clinical Site with 1.9K Patients	5	82	5.75%
Clinical Site with 1.8K Patients	6	91	6.19%
Clinical Site with 1K Patients	2	104	1.89%
**Insurance Class**			
Blue Cross Blue Shield	78	1863	4.02%
Commercial	61	1326	4.40%
Medicare	48	1105	4.16%
Medicare Managed	67	886	7.03%
Others	15	240	5.88%
Medicaid Managed	21	140	13.04%
**Race**			
White	268	5088	5.00%
Others	9	176	4.86%
Asian	1	128	0.78%
Hispanic	11	125	8.09%
Black	1	43	2.27%
**Language**			
English	285	5457	4.96%
Others	5	103	4.63%
**Charlson Deyo Score**			
1-2	63	1311	4.59%
3-4	130	2766	4.49%
>=5	97	1483	6.14%

**General Adult Score**			
0	64	1751	3.53%
1	105	2251	4.46%
2	59	1006	5.54%
3	28	372	7.00%
4	19	114	14.29%
5	11	35	23.91%
6	2	16	11.11%
7	1	9	10.00%
8	0	4	0%
9	0	2	0%
10	1	0	100%
**MyDH Active Within Last Year**			
Yes	259	5204	4.74%
No	31	356	8.01%
**Age Group**			
50-54	63	1003	5.91%
55-59	58	1081	5.09%
60-64	64	1236	4.92%
65-69	61	1245	4.67%
70-74	44	995	4.23%
**State Rank (ADI)**			
1	7	433	1.59%
2	15	410	3.53%
3	9	486	1.82%
4	17	564	2.93%
5	16	775	2.02%
6	38	747	4.84%
7	52	777	6.27%
9	48	512	8.57%
8	43	507	7.82%
10	45	349	11.42%
**Rural-Urban Commuting Area**			
1	42	1729	2.37%
2	8	295	2.64%
3	170	2143	7.35%
4	70	1392	4.79%

Supplementary Table S5: Consolidated Question Counts-- Attended

Category	Number Not Attended	Proportion Not Attended	Number Attended	Proportion Attended
**Unable to pay rent or mortgage in the past 12 months**				
No	276	0.0485	5414	0.9515
Yes	7	0.0886	72	0.9104
Patient Declined	2	0.0606	31	0.9377
Patient Refused	5	0.1042	43	0.8939
**Number of residences in the past 12 months**				
0	0	0.0	10	1.00
1	269	0.0476	5384	0.9524
2	21	0.1243	148	0.8751
>=3	0	0.0	18	1.0
**Unable to access medication due to transportation difficulties**				
No	279	0.0483	5494	0.9517
Yes	7	0.2059	27	0.7893
Patient Declined	4	0.093	39	0.905
**Feels isolated from others**				
Hardly ever	265	0.049	5148	0.951
Some of the time	13	0.0463	268	0.9536
Often	8	0.1111	64	0.8875
Decline	4	0.0476	80	0.9518
**Difficulty paying for necessities**				
Not Hard at All	222	0.0457	4632	0.9543
Not Very Hard	30	0.046	622	0.9539
Somewhat Hard	27	0.1195	563	0.858
Patient Declined	4	0.0635	59	0.9356
Hard	5	0.1613	26	0.8344
Very Hard	2	0.0833	22	0.9135
**Concerns about inadequate access to food in the past 12 months**				
Never True	273	0.0482	5393	0.9518
Sometimes True	9	0.1	81	0.9
Patient Declined	5	0.0704	66	0.9287
Often True	3	0.1304	20	0.8647

**Difficult reading medical material**				
Never	280	0.0495	5373	0.9505
Rarely	1	0.0139	71	0.9859
Sometimes	4	0.1176	30	0.8793
Decline	3	0.0667	42	0.932
Always	0	0.0	28	1.0
Often	2	0.1111	16	0.8834
**Lacked a consistent residence in the past 12 months**				
No	287	0.0496	5494	0.9503
Yes	3	0.0968	28	0.9004
Patient Refused	0	0.0	20	1.0
Patient Declined	0	0.0	18	1.0
**Unable to work due to lack of transportation**				
No	281	0.0487	5490	0.9513
Yes	6	0.1875	26	0.8078
Patient Declined	3	0.0638	44	0.9349
**Insufficient access to food in the past 12 months**				
Never True	276	0.0485	5411	0.9515
Sometimes True	8	0.1053	68	0.8935
Patient Declined	4	0.0548	69	0.9445
Often True	2	0.1429	12	0.8485

Supplementary Methods:

This section provides the detailed methodological framework underlying our Dartmouth Health case study, designed for broader application across diverse healthcare systems. The comprehensive framework encompasses data preprocessing strategies for handling SDoH variables, model selection and comparison approaches, performance evaluation protocols using nested cross-validation, and explainability analysis techniques. While our main manuscript focuses on the specific implementation within Dartmouth Health, this generalizable framework enables other healthcare organizations to adapt our approach to their own patient populations, data structures, and organizational contexts when developing SDoH-informed screening interventions.

Method M1: Data Pre-processing and Variable Construction

Our analytical process began by preprocessing three categories of predictor variables that shaped mammogram screening behavior: SDoH, demographic characteristics, and geographic factors, along with defining our outcome variables (scheduling and attendance status). We established a threshold for variable inclusion based on data completeness, excluding variables with excessive missingness (>80%). While higher than conventional thresholds, this approach was necessary to retain sufficient SDoH variables for analysis, given the inherently sparse nature of such data in clinical practice. We addressed potential concerns about data quality through our imputation strategy and sensitivity analyses using complete case analysis (Section 2.3). For standardization, we standardized SDoH questionnaire variables by merging duplicate questions that assessed similar constructs, retaining the question with more complete responses and consolidating the data. The SDoH questionnaire used in this study was developed internally by Dartmouth Health using selected questions from Epic's standard adult health screener modules, which assessed various social determinants including housing stability, food security, transportation access, and social support. We also converted inconsistent missing value representations including blank entries, 'N/A', 'NULL', and 'Unknown' responses to standardized missing values, while preserving meaningful response categories such as 'Patient Refused' and 'Choose not to disclose' as distinct categorical levels for analysis.

To facilitate a robust analysis of our demographic information, we consolidated language preferences into appropriate categories according to data availability and population distribution. We also combined race and ethnicity into major categories based on available data and sample size considerations. Similarly, we merged insurance type into primary groupings that reflected common coverage patterns. For geographic variables, we processed the Rural-Urban Commuting Area (RUCA) code and area deprivation index (ADI) to capture spatial dimensions of healthcare access and quantify neighborhood socioeconomic status. We removed the ADI national rank variable and retained only the ADI state rank for analysis. We also converted problematic ADI state rank codes including 'GQ' (Group Quarters), 'PH' (Other US territories), and 'QDI'

(Quality Data Issues) to missing values to ensure our data quality. Specific categorization details for our case study are described in Section 2.2.2.

Our framework assumed a binary outcome indicating whether a patient had scheduled a screening (1) or not (0), and another indicating whether a patient had attended their scheduled mammogram (1) or not (0). For the attendance framework, we only included patients who had a mammogram scheduled, determining attendance based on whether they received a mammogram within the past 27 months. This timeframe aligned with Dartmouth Health's internal clinical guidelines, which recommend women ages 50-75 with average risk receive mammogram screening every two years at a minimum, with a 27-month compliance window to account for scheduling delays. For the scheduling model, we included months since the last mammogram as a predictor variable; however, for the attendance model, we excluded this variable to avoid redundancy and potential data leakage since the 27-month timeframe was already incorporated into the attendance outcome definition. These screening statuses served as a direct indicator of patient engagement with screening guidelines and acted as the target variables for our predictive models.

To account for differences in patient health status that could influence screening behavior, we incorporated validated comorbidity measures as predictors in our machine learning models. The Charlson Comorbidity Index (Charlson *et al.*, 1987) provided a standardized measure of disease burden based on weighted scores for specific chronic conditions with demonstrated prognostic significance. This index had established excellent inter-rater reliability, high concurrent validity with other prognostic scales, and strong clinimetric sensitivity across diverse medical conditions (Charlson *et al.*, 2022). The Epic-derived General Adult Risk Score served as a comprehensive measure of medical risk based on patient age, recent healthcare utilization (e.g., acute admission, emergency visits), presence of chronic conditions (e.g., COPD, diabetes, heart failure, liver disease), depression status, primary care access, and insurance type; however, formal psychometric validation data for this specific algorithm had not been published in peer-review literature. Both measures were included as continuous variables in our predictive models as covariates to control for patient health complexity and comorbidity burden, allowing the algorithms to identify factors associated with screening behaviors independent of patient health complexity.

For both our scheduling and attendance analyses, we employed data imputation techniques using the missForest algorithm (Stekhoven and Bühlmann, 2012), a non-parametric imputation method based on random forests that can handle mixed-type data and complex interactions. This approach was necessary because relying solely on complete records would have substantially reduced sample sizes, which might have limited the robustness and generalizability of our models. We applied missForest separately to stratified training and testing sets to prevent data leakage randomly sampling the square root of the total number of variables in each tree of the

random forest and parallel processing to enhance computational efficiency. This imputation strategy enabled us to utilize information from a much larger patient population while addressing missing data in a principled way.

Method M2: Model Comparison

Our framework compared the performance of machine learning approaches for predicting a binary outcome of interest. For predicting mammogram scheduling, we compared elastic-net logistic regression (Zou and Hastie, 2005), random forest (Breiman, 2001), and light gradient boosting machine (Ke *et al.*, 2017). For predicting attendance, we selected different algorithms due to the smaller expected sample size (only patients who had scheduled mammograms), comparing Bayesian regression with prior log-odds based on previous literature (Gelman *et al.*, 2008), elastic-net logistic regression (Zou and Hastie, 2005), and decision tree classifier (Breiman, 2001). These machine learning techniques provided analytical strengths while maintaining interpretability for healthcare practitioners.

Method M3: Performance Evaluation

We developed a nested cross-validation scheme that incorporated 10 outer folds for model assessment and 5 inner folds for parameter optimization (Figure 1). This approach systematically tested how well our models would perform when applied to new patients not used in model development. By repeatedly training and testing on different patient subsets, this method provided a realistic estimate of model performance for healthcare systems implementing these tools in clinical practice.

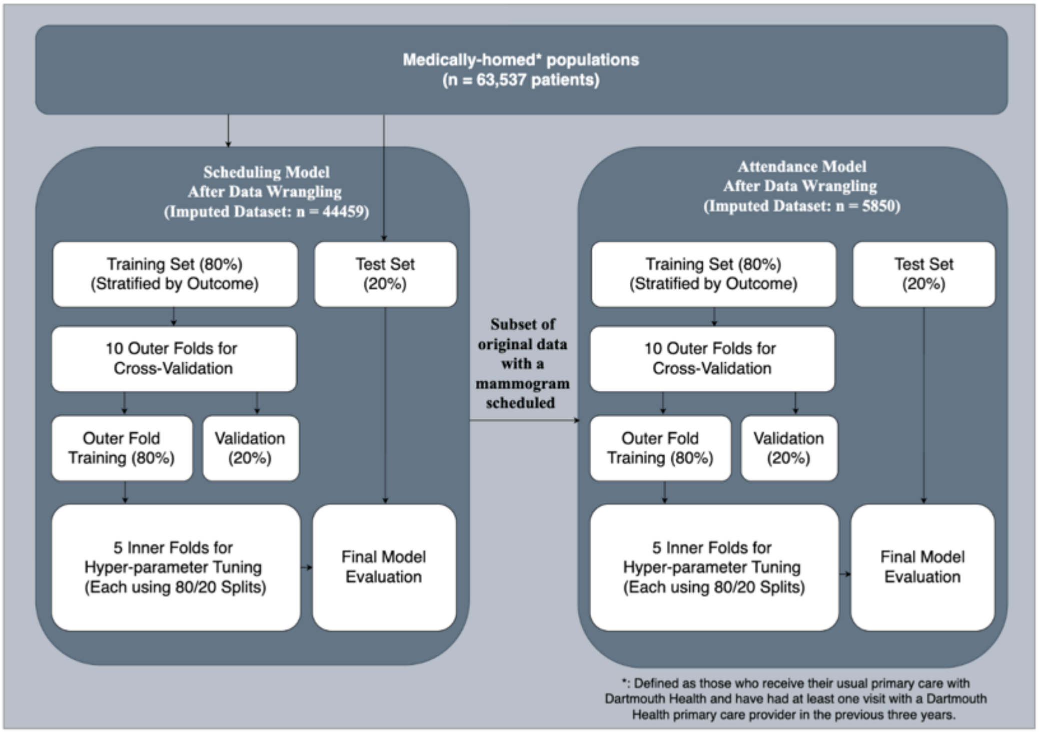

Figure M1: Data Splitting Process for both Scheduling and Attendance Model (Base Analysis with Imputed Data).

The data splitting process followed a stratified approach: we first divided the data into training (80%) and testing (20%) sets, then further divided the outer training data into inner training (80%) and validation (20%) subsets iteratively while maintaining the original distribution of mammogram scheduling behaviors (Varma and Simon, 2006). This structure allowed healthcare systems to assess how well the models might generalize across different patient populations and implementation contexts.

Our performance assessment utilized the Area Under the Receiver Operating Characteristic Curve (AUC-ROC) as the primary metric, chosen for its ability to evaluate model discrimination across different contexts (Sohil, Sohali and Shabbir, 2022). To address class imbalance challenges common in screening programs, we implemented the Random Over-Sampling Examples (ROSE) technique with synthetic information, ensuring our models effectively learned from both screened and unscreened populations (Lunardon, Menardi and Torelli, 2014).

To assess the statistical significance of performance differences between models, we conducted pairwise comparisons using DeLong's test for correlated ROC curves (DeLong, DeLong and Clarke-Pearson, 1988).

Method M4: Model Explainability Analysis

For the best-performing models, we conducted permutation importance analysis to measure the relative contribution of each predictor to the probability of mammogram scheduling and attendance (Altmann *et al.*, 2010). This analysis provided actionable insights for implementation strategies, measuring the relative contribution of each social driver to predicting non-screening behavior. The relationships between key predictors and screening behaviors were further visualized through partial dependence plots (Friedman, 2001), providing healthcare practitioners with intuitive tools for understanding how SDoH influenced screening behaviors in their specific implementation context.

Secondary Analyses:
Our analytical framework includes several secondary analyses designed to provide deeper insights into factors influencing mammogram screening behaviors. These analyses complement our primary models and offer healthcare systems actionable information for implementing targeted interventions.

Analysis A1: Scheduling Behavior
Supplementary Material S1.1: SDoH-Only Model
For scheduling behavior, we conducted a systematic exploration at multiple levels. First, we developed a SDoH-only model that isolates SDoH to understand their specific contribution to scheduling behaviors. This targeted approach helped identify which SDoH most significantly influence scheduling uptake, providing healthcare systems with evidence-based insights to design interventions that address the most influential social factors. Our analysis revealed that SDoH variables alone had limited predictive power (test AUC=0.51), with the model's nested cross validation yielding an average validation AUC of 0.51 and best performance of 0.54. The substantial performance gap between this specialized model and our comprehensive model (AUC=0.71) underscored the complex, inter-connected nature of factors influencing scheduling behavior.

Supplementary Material S1.2: Age-Stratified Analysis

Table S1.2: Age-Stratified Model Performance. Area Under the Curve (AUC) values and sample sizes across age groups for previously unseen test sets, demonstrating varying model performance across different age demographics.

Age Group	Average Validation AUC	Test Set AUC	Number of Patients in the Test Set
50-54	0.72	0.68	1658
55-59	0.7	0.7	1845
60-64	0.7	0.65	1928
65-69	0.71	0.67	1911
70-75	0.72	0.65	1550

We performed age-stratified evaluations of the light gradient boosting model across five-year intervals (i.e., 50-54, 55-59, 60-64, 65-69, 70-75) to identify how SDoH's influence varies across demographic groups. This stratification revealed relatively consistent predictive performance across different age groups (Table S1.2). The model showed minimal variation in performance, with average validation AUC values ranging from 0.7 to 0.72 across all age groups. The model performed slightly better for previously-unseen patients aged 50-54 (AUC=0.72, n=1342) and 70-75 (AUC=0.72, n=1212), compared to those aged 55-59 (AUC=0.7, n=1435) and 60-64 (AUC=0.7, n=1619), with the 65-69 age group falling in between (AUC=0.71, n=1505). This consistency suggested that social drivers of scheduling behavior may operate similarly across

different life stages, though the slight variations could still be considered when fine-tuning outreach strategies.

Supplementary Material S1.3: Patient-Level Models

To evaluate the independent contribution of individual characteristics to scheduling behavior, we developed a model focusing exclusively on patient-specific factors while excluding system-level variables. This individual-level model concentrated solely on patient characteristics (e.g., age, race, language, insurance class, months since the last mammogram, and ADI) and demonstrated relatively weaker predictive capability (test AUC=0.6) compared to our comprehensive model. The nested cross validation produced an average validation AUC of 0.65, with the best performance reaching 0.71 across validation folds. These results suggested that individual demographic and socio-economic factors provides limited explanatory power for scheduling behavior.

Supplementary Material S1.4: Clinic-Level Analysis

Our analysis of model performance by individual clinical site revealed substantial variation in predictive capabilities across different organizational settings (Table S1.4). The light gradient boosting machine model demonstrated strong performance in certain facilities, with Clinical Site with 1.8K Patients (average AUC=0.88, test AUC=0.73) and Clinical Site with 6.5K Patients (average AUC=0.74, test AUC=0.69) showing particularly robust predictive power. In contrast, other locations such as Clinical Site with 11K Patients (average AUC=0.66, test AUC=0.63) and Clinical Site with 4K Patients (average AUC=0.67, test AUC=0.67) exhibited modest performance. These facility-level variations in model effectiveness suggested that organizational contexts significantly influence the relationship between SDoH and scheduling behavior, highlighting the importance of organizational factors in implementation success.

Table S1.4: Clinical Site-Stratified Model Performance. Area Under the Curve (AUC) values across individual primary care practice locations, demonstrating how model performance varies across different organizational settings within the Dartmouth Health System.

Clinical Site (De-identified)	Average Validation AUC	Test Set AUC	Number of Patients in the Test Set
Clinical Site with 11k patients	0.66	0.63	1807
Clinical Site with 10.7k patients	0.7	0.65	1718
Clinical Site with 9.6k patients	0.69	0.65	1341
Clinical Site with 6.8k patients	0.74	0.65	964
Clinical Site with 6.5k patients	0.74	0.69	973
Clinical Site with 4k patients	0.67	0.67	697
Clinical Site with 2.2k patients	0.71	0.62	352
Clinical Site with 2k patients	0.68	0.57	315
Clinical Site with 1.9k patients	0.69	0.66	331
Clinical Site with 1.8k patients	0.88	0.73	265
Clinical Site with 1k patients	0.74	0.44	129

Supplementary Material S1.5: Complete Case Analysis

We conducted comparative analyses using complete cases only as a sensitivity check to evaluate how different approaches to missing data management affect implementation insights. The secondary analysis using complete case data demonstrated similar patterns but generally lower predictive performance. The logistic regression and random forest model achieved an average AUC of 0.662 and 0.685 respectively, while the light gradient boosting model showed comparable performance to the imputed data analysis (Average AUC=0.693). This consistency between complete and imputed analyses suggested that our findings are robust to different approaches for handling missing SDoH data, which is an important consideration for healthcare systems implementing SDoH scheduling programs.

The similar pattern of results between complete and imputed analyses, particularly in identifying key predictive factors, reinforced the reliability of our findings for implementation planning. The modest decrease in performance with complete case data suggested that the imputed SDoH data handling method may improve predictive accuracy, though the core relationships between social drivers and scheduling behavior remain stable across analyses.

Analysis S2: Attendance Behavior

Supplementary Material S2.1: SDoH-Only Attendance Model

We performed a similar SDoH-only analysis on attendance behavior. We developed a model that isolates the SDoH variables to comprehend their specific contribution to screening adherence, in particular the which SDoH most significantly impact screening adherence. Our aim with this analysis was to provide healthcare systems with powerful, evidence-based insights with the hope of creating interventions that address these social factors. Our isolated SDoH analysis, however, revealed that SDoH variables alone have limited predicted power (test AUC = 0.56). The model's nested cross validation had an average AUC of 0.55 with a best performance of 0.58. The marked performance gap relative to our full model (AUC = 0.69) emphasized the multifaceted and interrelated drivers of scheduling behavior.

Supplementary Material S2.2: Attendance Age Stratified Analysis

For our attendance analysis, we performed age-stratified evaluations of the logistic regression model across five-year intervals (i.e., 50-54, 55-59, 60-64, 65-69, 70-75) to explore how the impact of social determinants of health differs among various demographic groups. Unlike for scheduling behavior, the stratification of the attendance model revealed inconsistent performance across age groups (Table S2.2) and across our testing and validation sets. The model appeared to perform significantly better for patients aged 55-59 (AUC = 0.85, n = 239), than for those 50-54 (AUC = 0.67, n = 183), 60-64 (AUC = 0.68, n = 265), 65-69 (AUC = 0.68, n = 270), 70-75 (AUC = 0.40, n = 213). However, due to the large inconsistencies between the validation and testing performance, it is difficult to make any strong conclusions about the way in which SDoH

operate across different life stages. It is important to acknowledge that the small number of patients in each age group may contribute to the model's inconsistent performance.

Table S2.2: Age-Stratified Model Performance. Area Under the Curve (AUC) values and sample sizes across age groups for previously unseen test sets, demonstrating varying model performance across different age demographics.

Age Group	Average Validation AUC	Test Set AUC	Number of Patients in the Test Set
50-54	0.77	0.67	183
55-59	0.75	0.85	239
60-64	0.69	0.68	265
65-69	0.68	0.68	270
70-75	0.69	0.40	213

Supplementary Material S2.3: Attendance Patient Level Model

To assess how individual traits alone influence attendance behavior, we built a model that isolates patient-level factors, deliberately omitting system-related variables. This model included patient characteristics such as age, race, and ADI. The model demonstrated comparable predictive performance to our baseline model (AUC = 0.68). The nested cross validation had an average validation AUC of 0.69. These results suggested that patient-level characteristics alone carry substantial predictive power for attendance behavior, nearly matching the performance of more comprehensive models.

Supplementary Material S2.4: Attendance Clinical Level Analysis

Table S2.4: Clinical Site-Stratified Model Performance. Area Under the Curve (AUC) values

Clinical Site (De-identified)	Average Validation AUC	Test Set AUC	Number of Patients in the Test Set
Clinical Site with 11k patients	0.4	0.56	263
Clinical Site with 10.7k patients	0.64	0.67	252
Clinical Site with 9.6k patients	0.86	0.68	235
Clinical Site with 6.8k patients	0.58	0.85	87
Clinical Site with 6.5k patients	0.5	0.62	114
Clinical Site with 4k patients	0.35	0.75	70
Clinical Site with 2.2k patients	0.23	0.82	55
Clinical Site with 2k patients	0.5	0.5	26
Clinical Sites with 1.9k patients and 1.8k patients	0.63	0.55	46
Clinical Site with 1.0k patients	0.76	0.81	22

For our attendance analysis, we performed clinical site stratified evaluations of the logistic regression model to explore how the impact of SDoH differs across different clinics. Unlike for scheduling behavior, the stratification of the attendance model revealed inconsistent performance across the different clinic groups (Table S2.4) and across our testing and validation sets. Due to the large inconsistencies between the validation and testing performance, it is difficult to make any strong conclusions about the way in which SDoH operate across different clinical site. It is important to acknowledge that the small number of patients in each age group may contribute to the model's inconsistent performance. Due to this small number of patients, we also combined the results for the clinical site with 1.9k and 1.8k patients based on their geographic similarities.

Supplementary Material S2.5: Attendance Complete Case Analysis

We also conducted a complete case analysis for the attendance model as a sensitivity check. Secondary analysis on our complete data set for our chosen model (elastic-net logistic regression) showed comparable results to our model trained on the imputed dataset. Our logistic regression model trained on the complete dataset achieved an AUC of 0.716 which was comparable to our performance on the imputed dataset. However, we selected to use the imputed dataset because of its larger size to draw more meaningful insights.
